# 

**Published:** 1882-06-20

**Authors:** 


### EDITORIALS AND MISCELLANEOUS.

DON'T PUT IT OFF longer or forget to remit your back dues on subscription.

GO THOU AND DO LIKEWISE.—Dr. A. K. Davis writes: "It has just occurred to me that I have not paid for my Journal for last year, and now half the present year is gone. Accept the enclosed four dollars, and excuse me, for I have treated you badly." [Let all our friends who are in the same category, take counsel of Dr. D.'s good resolution and do as he did.—MANAGING EDITOR.]

SOUTHERN MEDICAL COLLEGE—See the advertisement of this Institution in the present number of this journal.

JEFFERSON MEDICAL COLLEGE.—We invite attention to the advertisement of the above Medical College in this journal.

PEOPLE'S CYCLOPEDIA.—Don't fail to read the advertisement of this great book and notice the proposition of the editor in connection with it.

DR. JNO. THAD. JOHNSON, one of the editors of the Atlanta Medical Register, has resigned his connection with that journal, which is now wholly conducted by Dr. J. B. Baird, of this city.

THE PHARMACOPŒIA.—The Publication of the revised Pharmacopœa has been awarded to Wm. Wood & Co., of New York. There bid was ten per cent. royalty, and a guarantee of the sale of 11,000 copies the first year.

THE SOUTHERN DENTAL JOURNAL, edited by B. H. Catching, D. D. S., and published in Atlanta, Ga. This is a beautiful and ably edited Journal. All men of the Dental profession in the South should take this Journal. We note in it many articles of value and interest to the Medical Practitioner.

CORRECTION.—Dr. Arch Dixon, author of the interesting article on Malarial Coma, in our May number, asks us to make the following corrections in said article. On page 167, nine lines from the bottom, read *with* pulmonary etc., and in the line below this instead of valuable *remedies* read valuable *time*.

TRANSACTIONS OF THE MICHIGAN STATE MEDICAL SOCIETY FOR THE YEAR 1881.—We have been favored with the above by Recording Secretary Geo. E. Ranney, M. D. Late but welcome. The following are the officers of the society for the ensuing year:

President—Dr. J. H. Jerome, of Saginaw city.

Vice President—Wm. T. Breakey, of Ann Arbor.

Secretary—Geo E. Ranney, of Lansing.

The report is creditable and contains a number of valuable and interesting papers.

The meeting for 1882 was held in Ypsilanti on the second Monday in May last.

### *RECEIPTED.*

1881.—Drs. D. S. Ellis, M. B. Willis, H. Thomas, R. L. Baker, E. N. Barton, H. H. Philpott, Robert L. Mason, T. H. Rallins, K. L. Minor, Jno. M. Strong.

1882.—Drs P. J. Parker, J. L. Hamilton, W. Jones, H. E. Archer, J. H. Henry, D. S. Aswell, W. T. Foute, Z. D. Emerson, D. R. Fox, T. S. Lyon, P. Taylor, E. A. Speed, J. B. Gordon, B. B. Dee, R. B. McCants, D. Maxwell, W. Jones, Charles G. McManaway.

